# Coupling coordination relationship between ecosystem services and water-land resources for the Daguhe River Basin, China

**DOI:** 10.1371/journal.pone.0257123

**Published:** 2021-09-10

**Authors:** Baodi Sun, Jingchao Tang, Dehu Yu, Zhiwen Song

**Affiliations:** 1 College of Architecture and Urban Planning, Qingdao University of Technology, Qingdao, China; 2 School of Environmental and Municipal Engineering, Qingdao University of Technology, Qingdao, China; 3 School of Civil Engineering, Qingdao University of Technology, Qingdao, China; Universidad de Murcia, SPAIN

## Abstract

Water and land resource utilization is an important driving force of changes in ecosystem services; therefore, research on multi-parameter coupling systems that consider “ecosystem services, water resources, and land resources” together has key significance for river basins. This study aims to reveal the interaction and mutual influence of ecosystem services and water and land resources in the Daguhe River Basin, China, based on the coupling coordination degree model. The results showed that during the period from 2000 to 2010, the coupling coordination degree values for the years 2000, 2005, and 2010 were 0.6005, 0.7292, and 0.8037. The corresponding coupling coordination classifications were categorized as “primary coordinated development”, “intermediate coordinated development,” and “well-coordinated development”, respectively. These results reflected the fact that the relationship between water and land resource utilization and the environment tends to evolve in the direction of coordinated development (an improvement in one part corresponds to an improvement in another part) with variation in water and land utilization types, and eventually pushes the whole resource, as well as ecological and environmental systems, from low to high levels of coupling coordination degrees as observed in case of the Daguhe River Basin, China. Our research provides an overview of the interaction between ecosystem services and water and land resources in the Daguhe Basin and even in the Shandong Province. With our results, we offer new perspectives on river basin management and for planning future eco-environmental policies (the policy is specifically designed for the ecological environment) by combining water and land resource utilization.

## Introduction

The ecosystem services in a river basin comprise its environmental conditions that are maintained for human survival and development and that are utilized in several ways by its inhabitants [1–3]. Ecosystem services are the foundation of human life and are closely related to anthropogenic welfare [4, 5]. With the development of urbanization and the continuous growth of populations, the water and land demand of the society has greatly increased, thus causing a degradation of water and land resources in river basins all around the world [6, 7]. Therefore, the contradiction between the well-being of the ecosystem and the utilization of water and land resources is becoming increasingly prominent.

The water and land resource utilization is an important driving force in changes in any ecosystem service, considering that it accounts for much of the human activities, such as water consumption and patterns of land use. The effects of water and land resource utilization in a river basin ecosystem can be summarized in two ways: (i) firstly, in the form of an impact on climate, soil, hydrology, and topography [4, 7, 8], (ii) secondly, through the change of eco-environmental factors (the factors or indicators from environment) and landscape patterns, which has a decisive influence on a regional ecosystem service [9]. In recent years, many researchers have focused on ecological water demand [10, 11], management models for water resources [12], spatial patterns of land use with regard to respect ecosystem services [13, 14], etc., while only a few to few studies have combined ecosystem services with to and water- land resources. Existing coupling relationship researches were only a simple correlation studies, such as Rost et al (2008) [15] quantified surface and groundwater to assess the impact of water resources on agricultural and non-agricultural terrestrial ecosystem services. Wang et al (2012) [16] studied the coupling relationship between land use pattern and ecosystem service value by simulating the structure of important ecological corridors. Guo (2016) [17] analyzed the effective relationship between water-land resources and ecosystem services, found different types of water and land resources had different effects on ecosystem services. The authors didn’t quantitatively calculate the coupling degree and coordination degree of coupling system for “ecosystem services, water resources, and land resources”. Shi et al (2021) [18] analyzed the effects of different future land use/land cover (LULC) scenarios on ecosystem services in the Yili River Valley, China by simulating the land-use changes during 2020–2030.

Research that considers multi-parameter coupling systems by involving ecosystem services and water and land resources together has key significance for studying ecosystem services in river basins. Coupling systems specifically refer to the coupling of ecological niches with changes taking place in space-time with limited water and land resources in river basins [19, 20]. The purpose of such coupling during studies is to ensure environmental development (the exploitation and protection for environment) and sustainable utilization of these resources and to ensure the maximization of ecological, social, and economic benefit. Research on such coupling systems, where we pay equal attention to ecology and economy, can help meet the goal of maximizing the ecological and economic benefits of land and minimizing the overall water shortage in the basin, so as to realize the optimal allocation of water and land resources in the river basin.

This study demonstrates coupling and coordination as a new perspective, where ecosystem services, water resources, and land resources are taken as three parts. Some differences exist between coupling and coordination, where coupling refers to the degree of interaction among the three parts [8, 21] without regard for advantages and disadvantages, while coordination shows the degree of coordination and reflects the benign coupling processes that take place among the three parts [21, 22]. This paper particularly focuses on the degree of coordination to analyze the coupling systems involving ecosystem services and water and land resources. From the perspective of coordination, the degrees of coupling and coordination determine the order and structure of the system in a critical region; however, they also help determine the tendency of the system to go from a state of disorder to a more ordered state. The function of the system, when combined quantitatively and qualitatively, can reflect the contribution of the fluctuation and change of each part to the evolution of the whole system [17, 19, 23]. In this paper, we attempt to investigate the interactions and the level of compatibility among the three parts outlined above using coupling coordination analysis in order to support a more coordinated development of the river basin ecosystem.

Moreover, due to the complexity and the scale of the river basin ecosystem, traditional methods of evaluation of the ecosystem services use case studies, which generally producing results with a high degree of uncertainty or error in predictions [3, 4, 24]. Therefore, we have referred to the transfer method described by a Chinese author named Gaodi Xie [25, 26], who put forth the equivalent factor method of scale transformation for evaluation. The equivalent factor method of ecosystem service evaluation combines the spatiotemporal variations in the river basin, which can then be analyzed for scale effects that combine the different water and land resources.

In this paper, we chose the Daguhe River Basin of Shandong Province in China as the study site. The Daguhe River basin plays an irreplaceable role in providing water and diverse ecosystems in the region. At present, research on the Daguhe River basin mainly focuses on ecological mechanisms, including the nutrient-carrying capacity of the water resources [27], as well as nitrogen and phosphate transport and transformation [28]. However, little research been conducted on the interactions between ecosystem services and water and land resources, particularly by incorporating effects on a spatiotemporal scale. Data from 2000 to 2010 was used to analyze the coupling coordination of ecosystem services and water and land resources.

The objectives of this study were to (1) evaluate ecosystem service values using the equivalent factor method; (2) calculate the matching coefficient of water and land resources; and (3) measure the coupling coordination degree between ecosystem services and water and land resource utilization. Our findings provide an overview of the interactional relationship between ecosystem services, water and land resources in Daguhe and even in the Shandong Province. These results will offer new perspectives for river basin management and for planning future eco-environmental policies that combine water and land resources.

## Materials and methods

### Study area and data

**Fig 1:**
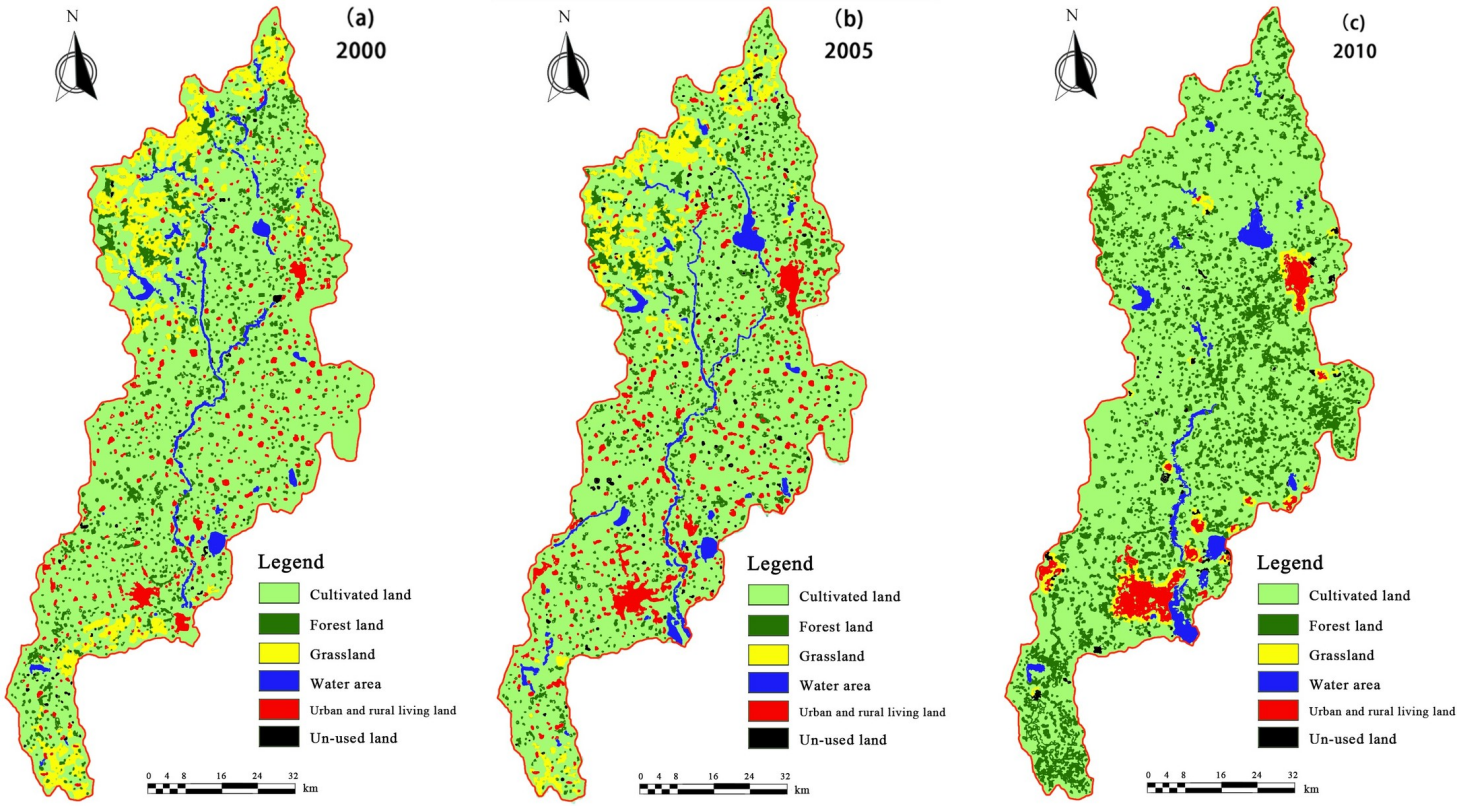
Distribution of land types over time.

The study area is located in the province of Shandong (E120°03′-120°25′, N36°10′-37°12′, China) and is named “Mother River” in the Qingdao city. It has a mean annual temperature of 12.30°C and a mean annual rainfall of 685.30 mm [29]. The total area is 62.05×10^4^ ha, and the total length of main stems is 199 km. The Daguhe River basin includes seven trunk streams, eight tributaries larger than 10,000 ha, and eight large and medium-sized reservoirs. Statistics on the change in different land utilization types from the year 2000 to 2010 are shown in Table 1 and Fig 1.

**Table 1:** Statistics on the change in different land utilization types over time (from aerial imagery and SPOT5 satellite imagery).

Land utilization types	Year 2000		Year 2005		Year 2010	
	Area (10^4^ ha)	Ratio (%)	Area (10^4^ ha)	Ratio (%)	Area (10^4^ ha)	Ratio (%)
Cultivated land	46.20	75.35	46.71	76.18	47.99	78.27
Forest land	1.59	2.60	1.59	2.60	3.63	5.92
Grassland	5.04	8.22	3.59	5.85	0.10	0.16
Water-covered area	1.93	3.14	2.59	4.23	2.26	3.68
Urban and rural construction land	6.38	10.41	6.81	11.10	6.55	10.69
Unused land	0.17	0.28	0.02	0.04	0.78	1.28
Total area	61.31	100.00	61.31	100.00	61.31	100.00

Data sources: The land use data were developed through remote sensing classification (from aerial imagery and SPOT5 satellite imagery) and field validation (classification accuracy: 93–95%).

Coupling coordination degree evaluation index system, including three parts: water resources, land resources, and ecosystem services, was employed in this study. Based on the Millennium Ecosystem Assessment and double counting [2, 30, 31], six ecosystem services included in this paper, which can be classified as provisioning, regulating, and cultural services. Provisioning service was mainly referred to substance production. Regulating services were referred to carbon sequestration, gas regulation, climate regulation, water purification. Cultural service was mainly referred to leisure tourism. Based on the types of land utilization and data sources, “land resources” refers to “land utilization”. Water resources mainly include agricultural water consumption, industrial consumption, domestic water, ecological water utilization, the total amount of water resources, water resources per unit area, the matching coefficient of water and land resources. Land resources include cultivated land, forest land, grassland, water-covered area, and urban and rural land.

Data used and resources were presented in Table 2. In this paper, we also used the related data on each districts the Daguhe River passed through for matching coefficient calculation, respectively, especially for ecosystem service evaluation based on the equivalent factor method, matching coefficient calculation of water and land resources, coupling coordination degree measurement between ecosystem services and water and land resource utilization.

**Table 2:** Data used and resources.

Three parts	Data resources
Land resources	1. The Land Resources and Planning Bureau of Qingdao
	2. Remote sensing classification (from aerial imagery and SPOT5 satellite imagery) and field validation (classification accuracy: 93–95%) [32, 33]
Water resources	1. Shandong provincial bureau of statistics
	2. Qingdao municipal bureau of statistics
Ecosystem services	Mainly referred to field test, interview survey, and socio-economic data.
	Field test including vegetation, water quality, soil, etc.
	Interview survey including travel expense survey.
	Socio-economic data including the amount of population, tourist arrivals, etc.

**Fig 2:**
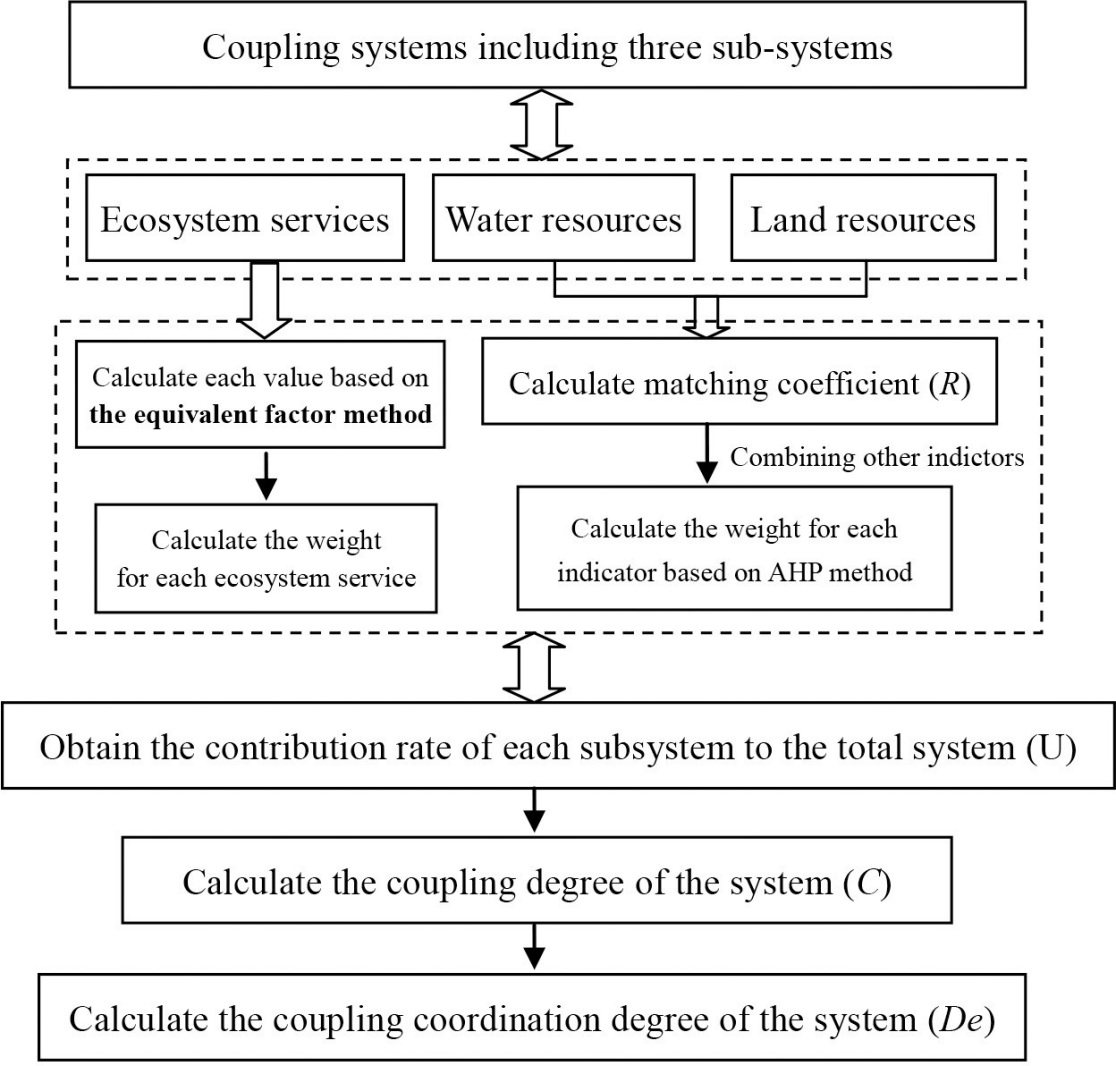
The flow chart shows the linkages between the different methodological approaches.

The linkages between the different methodological approaches included in this study can be shown on Fig 2. The methods mainly including the equivalent factor method, matching coefficient calculation model of water and land resources, the method for the contribution rate of each part, AHP (analytic hierarchy process) method and Coupling coordination degree measurements.

### Ecosystem service calculation

We referred to the method described previously by Xie et al (2015) [25] that was originally based on the work of Costanza (1997) [1] but had undergone some improvements; the net profit from grain production per unit area of farmland ecosystem was taken as one standard equivalent factor of the ecosystem service value. The grain yield value for farmland ecosystems was mainly calculated based on the three main grain products: rice, wheat, and corn. A multivariable model for one standard equivalent factor of each ecosystem service value was used as given in Eq 1:

$$D=S_{r}*F_{r}+S_{w}*F_{w}+S_{c}*F_{c}$$ (1)

Where *D* refers to one standard equivalent factor of an ecosystem service value (RMB/hm^2^; RMB is Chinese Yuan), and *S*_*r*_, *S*_*w*_, and *S*_*c*_ are percentages of the area planted with rice, wheat, and corn in the total area planted with the three crops in each year (%). Furthermore, *F*_*r*_, *F*_*w*_, and *F*_*c*_ are the average net profits per unit area of rice, wheat, and corn (RMB/ha). Data of this part obtained from Shandong Statistical Yearbook. The years evaluated in this study are 2000, 2005, and 2010, respectively. We built the equivalent value per unit area of different types of ecosystem services based on biomass (also called Net Primary Productivity, NPP) from remote sensing data and meteorological data, combining experts’ experiences and other published academic papers about ecosystem service evaluation. Biomass didn’t just reflect the capacity of substance production, at the same time, also had an important impact on other services during the process of the formation and accumulation for biomass (Xie et al., 2015) [25]. The specific calculation processes are as follows: First, we got the referred and adjusted equivalent value per unit area for each ecosystem services in Table 3. Second, we obtained one standard equivalent factor of an ecosystem service value (*D*) based on Eq 1. Third, we used D values multiply the related areas to calculate the ecosystem service values.

**Table 3:** The equivalent value per unit area for various ecosystem services.

Ecosystem classification	Providing services	Regulating services				Cultural services
	Substance production	Carbon sequestration	Gas regulation	Climate regulation	Water purification	Leisure tourism
Cultivated land	0.68	0.52	0.89	0.47	0.14	0.08
Forest land	0.42	2.32	1.91	5.71	1.67	0.93
Grassland	0.29	1.47	1.21	3.19	1.05	0.59
Water-covered area	1.03	0.93	0.77	2.29	5.55	1.89
Urban and rural construction land	0.00	0.00	0.00	0.00	0.00	0.00
Unused land	0.00	0.02	0.02	0.00	0.10	0.01

In socio-economic and geographical context, a positive effect of the income variable (GDP per capita) indicated that most farmland ecosystems had higher values in years with higher development levels [34]. Considering economic growth, the prices involved in ecosystem services should be adjusted to one standard year to compare. In this paper, the average net profits per unit area of rice, wheat, and corn (RMB/ha) need to be adjusted. In this way, we could only consider the changes of land area and ecological indicators resulting in the changes of the final ecosystem services in different years. The values of *D* in different years were all adjusted to the year 2010 using Eq 2 [4, 26].


$$D_{a}=\frac{X}{x_{i}}D_{i}$$ (2)

Where *D*_*a*_ is one standard equivalent factor of the ecosystem service value after adjustment by GDP; *X* is the average GDP of the Shandong Province, China in 2010; *x*_*i*_ is the average GDP of the Shandong Province, China in the original year evaluated (2000–2009); *D*_*i*_ is one standard equivalent factor of the ecosystem service value before adjustment by GDP in the original year evaluated (2000–2009).

### Calculation for water-land matching coefficient

Referring to the present literatures [17, 35], we found that the matching coefficient of water and land resources mostly based on agricultural water and land. We used the available water resources quantity per unit area of cultivated land to calculate the matching coefficients of water and land resources. We used the Daguhe River basin as a unit instance to calculate the matching levels of the agricultural water resources quantity and cultivated land areas. Therefore, the final model to calculate the matching coefficients of water and land resources was as follows:

$$R^{p}=\sum W_{p}\times a^{p}/\sum L_{p}$$ (3)

Where *R*^*p*^ is the matching coefficient for water and land resources (10^4^ m^3^/ha); *W*_*p*_ is the total available water resources quantity in the Daguhe River basin (10^8^ m^3^), *a*^*p*^ is the ratio of agricultural water resources quantity with respect to the total water quantity in the basin; *L*_*p*_ is the area of cultivated land in the basin (10^4^ ha).

The evaluation set of matching degrees for water and land resources is a collection of four grades. According to the values of *R*^*p*^, water and land resources matching degrees are divided into four grades: Grade Ⅰ—better matching level, Ⅱ—good matching level, Ⅲ—poor matching level, Ⅳ—very bad matching level; these correspond to *R* values of “*R*≥0.55”, “0.40≤*R*<0.55”, “0.25≤*R*<0.40”, “*R*<0.25”, respectively [19].

Meanwhile, we also used the related data on each district the Daguhe River passed through for matching coefficient calculation, respectively.

### Calculation for the contribution rate

The order parameter of three parts being considered in this paper, i.e., ecosystem services, water resources, and land resources, is denoted by U_*i*_ (*i* = 1, 2, 3). It indicates the contribution rate of each order parameter (each part) to the total system. The following formula was used to calculate U_*i*_:

$$U_{i}=\sum \lambda_{ij}\times x{’}_{ij}$$ (4)

Where λ_*ij*_ is the weight for the *j*th indicator of the *i*th order parameter, *x*_*ij*_(*j* = 1, 2,…, *n*) is the efficacy function after standardizing for the *j*th indicator (impact factor) of the *i*th order parameter; *x*’_*ij*_ is the order efficiency coefficient of the coupling system involving water and land resources and ecosystem services; *x*’_*ij*_∈(0, 1), where *x*’_*ij*_ represents the efficacy contribution values from *x*_*ij*_ to the coupling system. It further reflects the satisfaction degree of each impact factor to reach the target.

Moving on, *U*_*1*_, *U*_*2*_, and *U*_*3*_ are contribution values of each part to the total system order. We then calculated the value of *x*’_*ij*_ by using Eqs (5) and (6).


$$x{’}_{ij}=\frac{x_{ij}-\min(x_{ij})}{\max(x_{ij})-\min(x_{ij})}(\mathrm{the}\;\mathrm{positive}\;\mathrm{indicator})$$ (5)

$$x{’}_{ij}=\frac{\max(x_{ij})-x_{ij}}{\max(x_{ij})-\min(x_{ij})}(\mathrm{the}\;\mathrm{negative}\;\mathrm{indicator})$$ (6)

Where positive and negative indicators in Eqs (5) and (6), respectively, indicate that the larger the *x*’_*ij*_ values, the better was the positive indicator, meanwhile, negative indicators indicate that the smaller the *x*’_*ij*_ values, the better was the negative indicator. The matching coefficients (*R*) were included in *U*_2_ in this paper.

### Analytic hierarchy process method

λ_*ij*_ in Eq (4) was mainly calculated by combining AHP (analytic hierarchy process). The AHP method includes three steps [36, 37]. The first step is to establish a hierarchical model: we used 17 scores, from 1 to 9, plus their reciprocal values. The minimum value, 1/9, represented the lowest relative influence, while the highest value, a score of 9, represented the highest relative significance for stakeholders’ preferences for ecosystem services. In the next step, we evaluated the consistency of the ratings, which was done by calculating the consistency index (CI) and the consistency ratio (CR). The consistency index is defined by the equation: *CI* = $\frac{\lambda_{max}-n}{n-1}$, *CR* = *CI*/*RI*, where *λ*_max_ is the largest eigenvalue of a preference matrix and *n* is the number of parameters [38–40]. *RI* values have been tabulated by Saaty (1997) [36] as a function of *n*. Consistency ratios higher than 0.1 suggest untrustworthy judgments, indicating that the comparisons and scores should be revised. *λ*_max_ = ∑[(AW_i_)/nW_i_], W_i_ represents the eigenvector. We can calculate the weight of all factors at each level and ranking after the consistency check. Finally, we can make decisions according to the results of the rankings.

### Coupling coordination calculation for ecosystem services and water—land resources

Based on the concept of capacitance coupling and capacitance coupling coefficient model in physics, a coupling model including three parts has been established: water resources, land resources, and ecosystem services. It includes efficacy function, order parameter contribution, and coupling degree analysis. Finally, the coupling coordination degree can be calculated by the following formula:

$$C=m{\left\{\frac{U_{1}\times U_{2}\ldots U_{m}}{\Pi (U_{i}+U_{j})}\right\}}^{1/m}i=1,2,3,\ldots ,m$$ (7)

Where *C* is the coupling degree of the system, *C*∈(0, 1); m is the number of three parts, where m = 3 in this study. *U*_i_ and *U*_j_ mean any two among “*U*_1_, *U*_2_, *U*_3_”.

We introduced the coordination degree to quantitatively calculate the coupling degree of the three parts. We established coupling coordination degree function by using Eqs (8) and (9):

$$De={\left(C\times T\right)}^{1/2}$$ (8)

$$T=\alpha \times U_{1}+\beta \times U_{2}+\gamma \times U_{3}$$ (9)

Where *De* is the coupling coordination degree; *T* is the comprehensive coordination index of the three parts; α, β, and γ are the undetermined coefficients of each part’s contribution. The comprehensive coordination index could reflect the overall coordination effect of water and land resources and the ecosystem services in the river basin. Based on previous research by scholars [17, 34, 41], we considered α = 0.35, β = 0.35, γ = 0.30 in this study.

According to the values of *De*, the classification of coupling coordination degree includes six aspects (Table 4).

**Table 4:** Classification of coupling coordination degree.

Level	Classifications of coupling coordination degree	The range of *De* values
Ⅰ	High-quality coordinated development	0.90≤*De*≤1.00
Ⅱ	Well-coordinated development	0.80≤*De*<0.90
Ⅲ	Intermediate coordinated development	0.70≤*De*<0.80
Ⅳ	Primary coordinated development	0.60≤*De*<0.70
Ⅴ	Barely coordinated development	0.50≤*De*<0.60
Ⅵ	Threatened recession	*De*<0.50

Data sources: Comprehensive references to the existing results from Lv et al (2013).

In this paper, *C* refers to the coupling of ecological niches with changes taking place in space-time with limited water and land resources in river basins, reflecting the degree of interconnectedness and dependence between ecosystem services and water-land resources. *De* refers to the degree of benign coupling in interaction of the three parts (ecosystem services, water resources, land resources). we constructed the coupling coordination function to reflect the overall coordination effect of ecosystem services, water resources and land resources.

## Results

### Ecosystem service values evaluated by the equivalent factor method

According to the data in Table 2 and land areas for each type, we can calculate the equivalent values of each ecosystem service for different years. Based on the data thus acquired on land utilization and from the Shandong province statistical yearbooks for the years 2000, 2005, and 2010, Eqs (1) and (2) from Section 2.1 were combined, and the *D* (one standard equivalent factor of ecosystem service) values for each year: 2000, 2005, and 2010, were calculated to be 283708.71, 353202.26, and 382264.47 RMB/ha, respectively. We could finally calculate the total economic values of different ecosystem services for the different years (Table 5). There was an increase of approximately 32.55% over the period between 2000 and 2010. This increase was primarily because of an increase in cultivated land, forest land, and water-covered area, which increased from 75.35% to 78.27%, 2.60% to 5.92%, 3.14% to 3.68%, respectively over this time period. The ecosystem services of substance production, carbon sequestration, gas regulation, etc. can especially be increased for cultivated and forest land [42].

**Table 5:** The total economic values of different ecosystem services for different years (Chinese Yuan: RMB).

Years	Substance production	Carbon sequestration	Gas regulation	Climate regulation	Water purification	Leisure tourism	Total
2000	1.01*10^7^	1.05*10^7^	1.47*10^7^	1.46*10^7^	0.18*10^7^	0.33*10^7^	5.50*10^7^
Ratio	18.33%	19.05%	26.70%	26.50%	3.34%	6.08%	100.00%
2005	1.28*10^7^	1.26*10^7^	1.80*10^7^	1.71*10^7^	0.23*10^7^	0.43*10^7^	6.71*10^7^
Ratio	19.02%	18.78%	26.82%	25.50%	3.44%	6.44%	100.00%
2010	1.40*10^7^	1.36*10^7^	1.97*10^7^	1.86*10^7^	0.26*10^7^	0.44*10^7^	7.29*10^7^
Ratio	19.15%	18.68%	27.02%	25.57%	3.53%	6.05%	100.00%

When compared the periods of 2000–2005 and 2005–2010, to find out that the total values of ecosystem services increased approximately by 22.00% and 8.64%. Due to additions in cultivated land and water-covered area, the values for 2000–2005 showed a larger increase than those for 2005–2010. On specific analysis of each ecosystem service, we observed that substance production, carbon sequestration, gas regulation, and climate regulation had faster growth in 2000–2005 than in 2005–2010.

Meanwhile, the economic values of different ecosystem services for eight regions in 2000, 2005, 2010 were shown on S1–S3 Tables.

### Matching coefficient calculation of water and land resources

We calculated the matching coefficient of water and land resources by taking into account the average quantity of water used in agriculture over the years and the area of cultivated land in related years for the Daguhe River Basin; this information was then combined to calculate the matching coefficient calculation model. The matching degrees of water and land resources were spatiotemporally analyzed. Matching coefficient calculation for time was done by including the years 2000, 2005 and 2010. Matching coefficient calculation for space covered eight different districts that the Daguhe River passes through, including Zhaoyuan, Pingdu, Laixi, Jimo, Chengyang, Jiaozhou, Gaomi, and Xihai’an.

**Fig 3:**
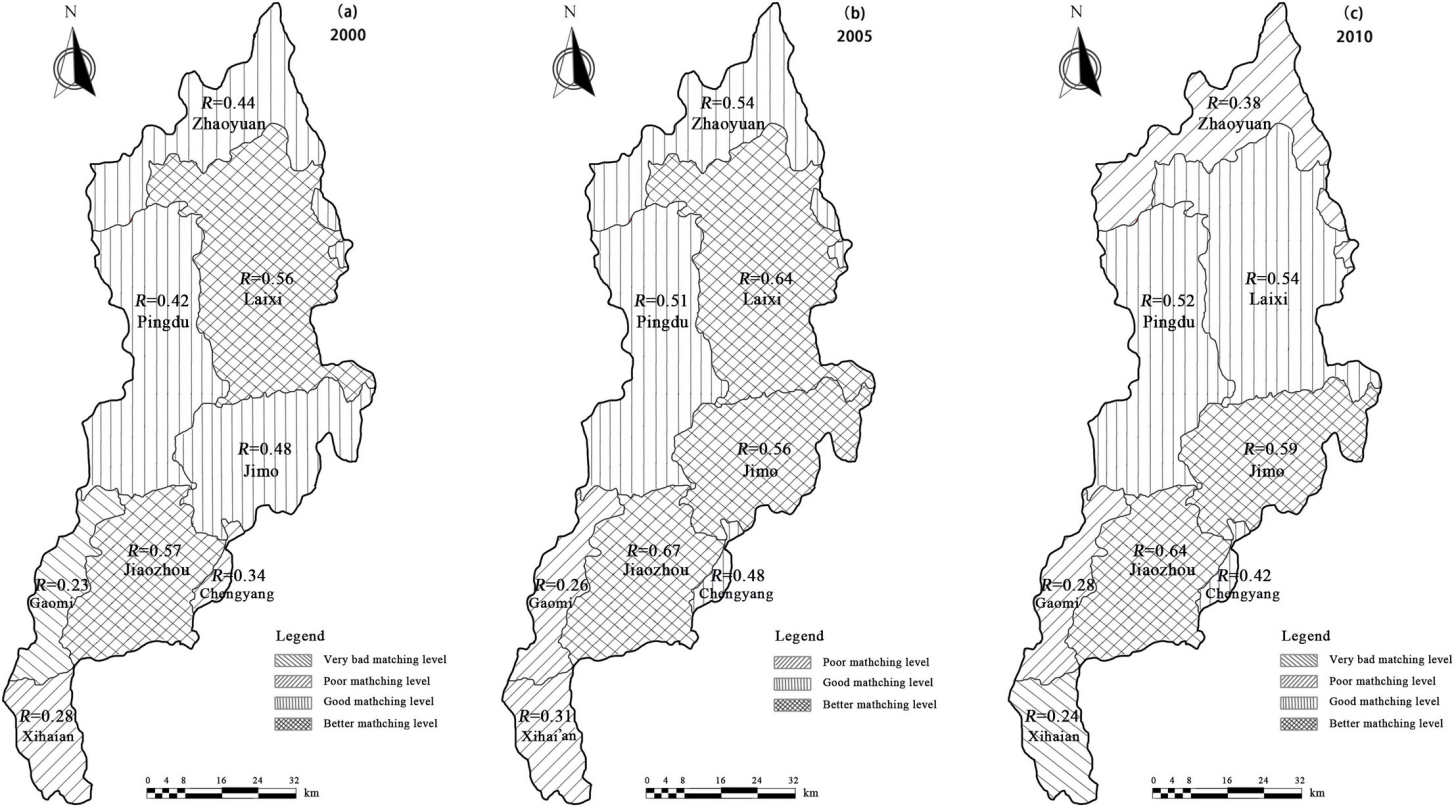
The matching levels of different space districts in different years.

The matching coefficient results for water and land resources in each district are shown in Fig 3. Based on the *R* values, different time and space presented a different matching degree. The *R* values for the years, 2000, 2005, and 2010, were 0.43, 0.61, and 0.53, respectively. The *R* values might go up and then down with the total available water resources and the ratio of agricultural water resources both go up and then down for the years, 2000, 2005, and 2010. These results indicated that the water and land resources fell in level Ⅱ (good matching level), level Ⅰ (better matching level), and level Ⅱ (good matching level). A comparison of (a), (b), and (c) in Fig 3 revealed that the *R* values (the matching coefficient for water and land resources) in the year 2005 was relatively higher than in the years 2000 and 2010. There were three districts in level Ⅰ (better matching level), three districts in level Ⅱ (good matching level), two districts in level Ⅲ (poor matching level), and no district lied in level Ⅳ (very bad matching level).

Moreover, the levels of space matching in different years can also be seen. Most districts had different matching levels in different years, except for Pingdu district that lied in level Ⅱ (good matching level) in all years and the Jiaozhou district that lied in level Ⅰ (better matching level) in the three years. The water and land resource matching levels of Zhaoyuan district were in Ⅱ (good matching level) in the years 2000 and 2005 and in level Ⅲ (poor matching level) in the year 2010. The matching levels of Laixi district were in level Ⅰ (better matching level) in 2000 and 2005 and in level Ⅱ (good matching level) in 2010. The matching levels of Xihai’an district were in level Ⅲ (poor matching level) in 2000 and 2005 and in level Ⅳ (very bad matching level) in 2010.

The matching levels of Zhaoyuan, Laixi, and Xihai’an declined with time. However, the levels for the other three districts, Jimo, Chengyang, and Gaomi, increased with time. The matching levels of Jimo lied in level Ⅱ (good matching level) in 2000 and in level Ⅰ (better matching level) in 2005 and 2010. The Chengyang district had level Ⅲ (poor matching level) in 2000, and level Ⅱ (good matching level) in 2005 and 2010, while the Gaomi district had level Ⅳ (very bad matching level) in 2000 and level Ⅲ (poor matching level) in 2005 and 2010.

### The contribution rate of each indicators of three parts

Based on the calculation of ecosystem service values and the water and land resource matching levels, Equs of (4)–(9) were combined so we could measure the coupling coordination degree among the three parts. Moreover, by using Eqs (5) and (6), we standardized the data on ecosystem services and land and water resources. Evaluation index weights (λ_*ij*_) in Eq (4) were then calculated by combining AHP (analytic hierarchy process) [43, 44]; their values are given in Table 6.

**Table 6:** Weight of the evaluation indexes.

Three parts	Index names	Index codes	Index types	Weights
Ecosystem service (1.0000)	Substance production	X1	+	0.1915
	Carbon sequestration	X2	+	0.1868
	Gas regulation	X3	+	0.2702
	Climate regulation	X4	+	0.2557
	Water purification	X5	+	0.0353
	Leisure tourism	X6	+	0.0605
Water resource (1.0000)	Total amount of water resource	X7	+	0.2931
	Water resources per unit area	X8	+	0.1620
	The matching coefficient of water and land resources	X9	+	0.2398
	The ratio of agricultural water consumption	X10	−	0.1963
	The ratio of industrial consumption	X11	−	0.0622
	The ratio of domestic water	X12	−	0.1072
	The ratio of ecological water utilization	X13	−	0.0094
Land resource (1.0000)	The ratio of cultivated land	X14	+	0.2402
	The ratio of forest land	X15	+	0.2065
	The ratio of grassland	X16	+	0.1728
	The ratio of water-covered area	X17	+	0.2204
	The ratio of urban and rural land	X18	−	0.1601

Note: these indexes are also called *x*_ij_, “+”, “—” represent the positive and negative indicators, respectively, in the coupling coordination system. Their efficacy functions are based on Eqs (5) and (6).

In the “ecosystem service” part, among all six indexes, gas regulation and climate regulation accounted for the largest weight as compared to all other indexes. The weights of these two indexes were 0.2702 and 0.2557, while the other four indexes had weights lesser than 0.2000. Water purification carried the smallest weight in the overall part, i.e., 0.0353. In the “Water resource” part, the total amount of water resource accounted for the largest weight of 0.2931, followed by the matching coefficient of water and land resources (0.2398), the ratio of agricultural water consumption (0.1963), and the ratio of domestic water (0.1072). The ratios of industrial consumption and of ecological water utilization accounted for relatively smaller weights that were only 0.0622 and 0.0094. In the “land resource” part, the ratios of cultivated land, water-covered area, forest land, grassland, and urban and rural land carried weights of 0.2402, 0.2204, 0.2065, 0.1728, and 0.1601, respectively.

### Coupling coordination degree measurement between ecosystem services and water and land resource utilization

According to these results, by combining the values of the order parameter of each part (*U*_1_, *U*_2_, *U*_3_), we can calculate the coupling degree (*C*) and the coupling coordination degree of the system (*De*) (Table 7). The values of *C* for years, 2000, 2005, and 2010, were 0.8542, 0.9037, and 0.8960. The values of *De* for years, 2000, 2005, and 2010, were 0.6005, 0.7292, and 0.8037, respectively. The coupling coordination classifications for these three years were “primary coordinated development”, “intermediate coordinated development,” and “well-coordinated development”.

**Table 7:** Coupling coordination results for different years.

Years	*U*_1_	*U*_2_	*U*_3_	*C*	*De*	Coupling coordination classification
2000	0.5013	0.5017	0.3025	0.8542	0.6005	Primary coordinated development
2005	0.5440	0.6543	0.5271	0.9037	0.7292	Intermediate coordinated development
2010	0.6306	0.7428	0.7219	0.8960	0.8037	Well-coordinated development

Note: *U*_1_, *U*_2_, and *U*_3_ represent the order parameter of each part: ecosystem services, land resources, and water resources, respectively. These were calculated using Eq (4). Here, *C* represents the coupling degree and *De* represents the coupling coordination degree of the system.

**Fig 4:**
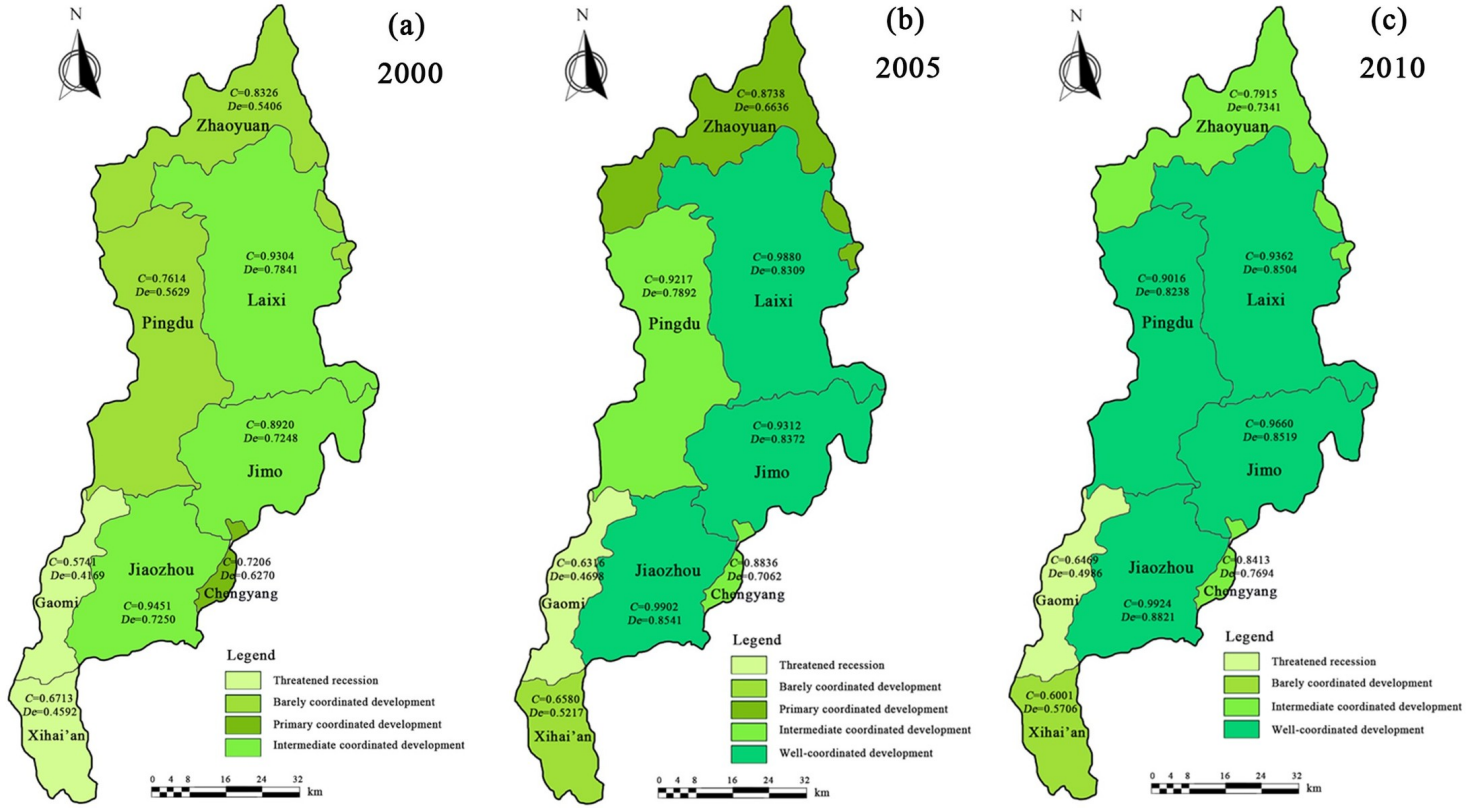
Coupling coordination results for the ecosystem services and water and land resource utilization for different spatial districts.

Moving on, we can further calculate the coupling coordination results of ecosystem services and water and land resource utilization for different spatial districts (Fig 4).

The coupling coordination degree values of all spatial districts presented an increasing trend of the levels with time, i.e., more and more spatial districts were present in “well-coordinated development” status (two in 2000, three in 2005, and four in 2010), while lesser spatial districts were present in “threatened recession” status (two in 2000, one in 2005 and 2010) each. Furthermore, the value of *De* in 2010 was higher than that in 2005.

## Discussion

In this paper, we used coupling coordination degree to quantitatively evaluate the Daguhe River basin from the year 2000 to 2010. We performed a combined analysis of the spatiotemporal coupling coordination degree to promote method innovation and breakthroughs in coupling coordination theories. Our research is a vital step in developing a multi-scale explanatory framework to relate ecosystem services with water and land resource utilization. What we actually contributing are: we offer new perspectives on river basin management and for planning future eco-environmental policies (the policy is specifically designed for the ecological environment) by combining water and land resource utilization.

### Analysis of spatiotemporal coupling coordination degree

From the perspective of the total coupling of a system, the coupling coordination degree presents better status of a system with respect to time. This reflects the fact that the relationship between water and land resource utilization and the environment evolves in the direction of coordinate development and eventually pushes the whole resource and ecological system from low to high levels of coordination [45–48]. The emergence of the above phenomenon resulted from recent ecological restoration and policies for Daguhe River Basin, such as decision–support system established for flood control [49]; exploration and innovation of ecological governance models involved in theories of “water security, water resources, water environment, water ecology, water culture” [50, 51]; much attention be paid to surface water and groundwater by using numerical simulation models combining analysis of ecological carrying capacity [52] in Daguhe River Basin.

According to the analysis of spatial districts in different years, the ecosystem service and coupling coordination degree values showed increasing trends, along with the matching coefficient of water and land resources, followed by a slow decrease. The overall degree of coupling coordination for the three parts analyzed here: ecosystem service, water resources, and land resources, was not only related to the matching coefficient of water and land resources, but also to the ecosystem service values [53, 54]. Based on the classifications for land type, cultivated land and forest land increased from the year 2000 to 2010; therefore, many ecosystem service values contributed by them, such as carbon sequestration, substance production, etc., also increased [14, 55–57]. Considering the different results for each spatial district, we combined the water and land resources to clarify these differences. Most water-covered areas were distributed in Jiaozhou, Jimo, and Laixi, and thus, the coupling coordination degrees of these three districts were higher than those for others.

In corroboration of our findings, Lv et al (2013) [19] and Vollmer et al (2018) [58] also pointed out that the distribution of water-covered areas had obvious relativity between coupling coordination systems involving the ecosystem and water resources. However, lesser water-covered areas were present in Xihai’an and Gaomi districts, therefore, their coupling coordination degrees were also lesser than others, especially the Gaomi district always lied in the “threatened recession” status. In addition, the matching coefficient of water and land resources in Gaomi district were present in level Ⅳ (very bad matching level) in 2000 and in level Ⅲ (poor matching level) in 2005 and 2010, which indicated that cultivated land and agricultural water consumption were essential for local water and land resources, which can further affect the function of ecosystem services [59].

### Comparisons with other similar studies

In recent years, two similar studies had representativeness from the authors of Lv (2013) [19] and Guo (2016) [17]. Lv calculated the coupling degree and coordination degree of the system for “ecological environment assessment, water resources, and land resources”. Guo analyzed the effective relationship between water-land resources and ecosystem services, but she didn’t calculate the coupling degree and coordination degree of coupling system for “ecosystem services, water resources, and land resources”. This paper combined the above two studies, demonstrated “coupling and coordination” as a new perspective, where “ecosystem services, water resources, and land resources” were taken as three parts. In recent researches, there are also some other studies such as Xu et al (2020) [45] combining water-land use efficiency with economic development together to achieve the sustainable utilization of water-land resources and the sustainable development of the economy. It can be seen that human beings are paying more and more attention on the relationship between water-land resources and the environment as well as the development of the whole city.

Therefore, what’s new and interesting about this paper is we regarded three parts of “ecosystem services, water resources, land resources” as a whole, successively by ecosystem services evaluation, calculation for water-land matching coefficient, Coupling coordination calculation for ecosystem services and water—land resources, then further comprehensively understanding the eco-environmental status of the overall or each spatial districts for the Daguhe river basin. Meanwhile, it will bring up a new question about whether our findings apply to other similar river basin; especially experience the same type of land conversion with this paper. From our study, we knew the ecosystem service values (per unit) of forest land were significantly higher than grassland, which was the main driver for the coupling coordination degree values increasing. More specifically, variations of water—land resources types result in variations of ecosystem services values, further lead to variations of the whole coupling coordination degree. Therefore, we would expect different results for types of other basins owned other variations of water—land resources types.

Nevertheless, there is still space for further improvements in the modeling establishment due to the limited data (three time nodes of 2000, 2005 and 2010) and incomplete store of some related professional knowledge in this research. Firstly, indicators selection in the three subsystems of “ecosystem services, water sources, and land sources” can be improved through some theoretical analysis rather than subjective selection. For example, a driving force analysis could be used to explore some key factors influencing the “ecosystem services—water sources—land sources” systems, which will produce a more reliable result in the future study. More indicators, such as degree of land use (comprehensive index of land use degree), land use benefits (gross agricultural output value per capita; grain output per capita) in subsystem of “land sources”, utilization rate of water resources in subsystem of “water sources”, are suggested to be considered in the system of evaluation indexes (Table 6 in this paper). Secondly, the contribution coefficients α, β and λ in the coupling coordination degree function by the formula 9 in this research are usually defined according to the previous researches and some related professional knowledge, which may lead to the uncertainty and distortion in the final evaluation results due to subjectivity. Therefore, a new calculation and definition of the contribution coefficients obtained by using the synergy theory can be considered in future studies [60, 61]. Thirdly, the ecosystem services valuation could be overestimated in our study. Generally, the ecosystem service valuations are long-term/equilibrium valuations, whereas the changes in this study are relatively short-term. For example, the forested area doubled in the last 5 years of the study. A 5-year-old forest doesn’t have the same effects on carbon sequestration, water purification, etc. as of a mature forest [1, 25, 26]. It will be a new direction to calculate the accurate ecosystem service values based on different forest ages and types in future studies. Lastly, there is evidence that the indicators or factors and their dynamic coupling processes in other coupling systems such as the coupling coordination between urbanization and eco-environment have spatial interaction [62, 63]. Thus, the process of modeling establishment could take some other social factors into consideration to generate a more accurate and scientific evaluation result of ecosystem services-water sources–land sources function.

## Conclusion

In this paper, the ecosystem service values increased by approximately 32.55% from 2000 to 2010, revealing that the variation in land types was the main driving force of ecosystem services. According to the coupling coordination degree values and the matching coefficient of water—land resources, we could further comprehensively understand the eco-environmental status of each spatial district for the Daguhe river basin. We should pay more attention on protection measurements for the districts owned higher values, and pay more attention on ecological restoration to improve the function for the districts owned smaller values.

Last but not least, the final coupling coordination degree values for the years, 2000, 2005, and 2010, were 0.6005, 0.7292, and 0.8037, which reflect the fact that the relationship between water—land resources and ecosystems tends toward evolving in the direction of coordinate development with variation in water—land resources types. Drivers behind these changes may were variations of water—land resources types, further may result in variations of ecosystem services values. It could offer new perspectives on planning future eco-environmental policies for river basin mangers and researchers.

## Supporting information

S1 Table The economic values of different ecosystem services for eight regions in 2000 (Chinese Yuan: RMB). (DOCX)

S2 Table The economic values of different ecosystem services for eight regions in 2005 (Chinese Yuan: RMB). (DOCX)

S3 Table The economic values of different ecosystem services for eight regions in 2010 (Chinese Yuan: RMB). (DOCX)

## References

[1] Costanza R, dArge R, deGroot R, Farber S, Grasso M, et al. The value of the world’s ecosystem services and natural capital. Nature. 1997; 387: 253–260.

[2] A M. Ecosystems and human well-being. Washington. DC: Island Press. 2005.

[3] Chaikumbung M, Doucouliagos H, Scarborough H. The economic value of wetlands in developing countries: A Meta-regression analysis. Ecol. Econ. 2016; 124: 164–174.

[4] Sun BD, Cui LJ, Li W, Kang XM, Pan X, et al. A meta-analysis of coastal wetland ecosystem services in Liaoning Province, China. Estuar. Coast. Shelf. S. 2018; 200: 349–358.

[5] Sun BD, Tang JC, Yu DH, Song ZW, Wang PG. Ecosystem health assessment: A PSR analysis combining AHP and FCE methods for the Jiaozhou Bay, China. Ocean. Coast. Manage. 2019; 168: 41–50.

[6] Smiraglia D, Ceccarelli T, Bajocco S, Salvati L, Perini L. Linking trajectories of land change, land degradation processes and ecosystem services. Environ. Res. 2016; 147: 590–600. doi: 10.1016/j.envres.2015.11.030 26654561

[7] Voss BM, Wickland KP, Aiken GR, Striegl RG. Biological and land use controls on the isotopic composition of aquatic carbon in the Upper Mississippi River Basin. Global. Biogeochem. Cy. 2017; 31: 1271–1288.

[8] Burcher CL, Valett HM, Benfield EF. The Land-Cover Cascade: Relationships Coupling Land and Water. Ecology. 2007; 88: 228–242. doi: 10.1890/0012-9658(2007)88[228:tlcrcl]2.0.co;2 17489471

[9] Yi H, Güneralp B, Kreuter UP, Güneralp I, Filippi AM. Spatial and temporal changes in biodiversity and ecosystem services in the San Antonio River Basin, Texas, from 1984 to 2010. Sci. Total. Environ. 2018; 619: 1259–1271. doi: 10.1016/j.scitotenv.2017.10.302 29734604

[10] Rost S, Gerten D, Bondeau A, Lucht W, Rohwer J, et al. Agricultural green and blue water consumption and its influence on the global water system. Water. Resource. Res. 2008; 44: 137–148.

[11] Wu X, Zheng Y, Wu B, Tian Y, Han F, et al. Optimizing conjunctive use of surface water and groundwater for irrigation to address human-nature water conflicts: A surrogate modeling approach. Agr. Water. Manage. 2016; 163: 380–392.

[12] Rockström J, Karlberg L, Wani SP, Barron J, Hatibu N, et al. Managing water in rainfed agriculture—the need for a paradigm shift. Agr. Water. Manag. 2012; 97: 543–550.

[13] Barros C, Guéguen M, Douzet R, Carboni M, Thuiller W. Extreme climate events counteract the effects of climate and land-use changes in Alpine tree lines. J. Appl. Ecol. 2016; 54: 39–50. doi: 10.1111/1365-2664.12742 28670002 PMC5489083

[14] Gashaw T, Tulu T, Argaw M, Worqlul AW, Tolessa T, et al. Estimating the impacts of land use/land cover changes on Ecosystem Service Values: The case of the Andassa watershed in the Upper Blue Nile basin of Ethiopia. Ecosyst. Serv. 2018; 31: 219–228.

[15] Rost S, Gerten D, Bondeau A, Lucht W, Rohwer J, Schaphoff S. Agricultural green and blue water consumption and its influence on the global water system. *Water Resources Research*, 2008, 44(9): 137–148.

[16] Wang J, Li F, Qian Y, Yin C X. Landscape security pattern design based on ecosystem service. *Environmental Science & Technology*, 2012, 35(11): 199–204.

[17] Guo Y. Research on optimal allocation of water and soil resources for ecosystem services. Zhengzhou. University. Doctoral dissertation. 2016.

[18] Shi M J, Wu H Q, Fan X, Jia H T, Dong T, He P X, et al. Trade-Offs and Synergies of Multiple Ecosystem Services for Different Land Use Scenarios in the Yili River Valley, China. *Sustainability*, 2021, 13, 01577.

[19] Lv J, Yuan XP, Gan S, Yang ML, He Q, et al. Research on Evaluation and Development Potential Analysis of Low-Slope Hilly Land Resources. Appl. Mech. Materials. 2013; 444–445: 1260–1264.

[20] Ji C, Zhan QW, Hong WZ. Integrated Evaluation of Coupling Coordination for Land Use Change and Ecological Security: A Case Study in Wuhan City of Hubei Province, China. Inter. J. Environ. Res. Pub. Heal. 2017; 14: 1435. 10.3390/ijerph14111435 PMC5708074 29165365

[21] Pinder RA, Renshaw I, Davids K. Information–movement coupling in developing cricketers under changing ecological practice constraints. Hum. Movement. Sci. 2009; 28: 468–479. doi: 10.1016/j.humov.2009.02.003 19339072

[22] Moumita M, Jinu J, Joyanta C. Coordination-polymer anchored single-site ‘Pd-NHC’ catalyst for Suzuki-Miyaura coupling in water. J. Chem. Sci. 2018; 130: 83.

[23] Fang CL. Basic laws of the interactive coupling system of urbanization and ecological environment. Arid. Land. Geograph. 2006; 2: 1–8.

[24] Sabatino AD, Coscieme L, Vignini P, Cicolani B. Scale and ecological dependence of ecosystem services evaluation: spatial extension and economic value of freshwater ecosystems in Italy. Ecol. Indic. 2013; 32: 259–263.

[25] Xie GD, Zhang CX, Zhang LM, Chen WH, Li SM. Improvement of the evaluation method for ecosystem service value based on Per Unit Area. J. Nat. Res. 2015; 30: 1243–1254.

[26] Xie GD, Zhen L, Lu CX, Xiao Y, Chen C. Expert knowledge-based valuation method of ecosystem services in china. J. Nat. Res. 2008; 23: 911–919.

[27] Lu HY, Li Q, Xie XM, Wang J, Kang AQ. Research on water resources carrying capacity of Daguhe River Basin in Qingdao city. Water. Resource. Power. 2011; 29: 21–24.

[28] Ma XB, Yin ZG, Sun YJ, Wang ZY. Nitrogen, phosphate transport and transformation research at Dugu Estuary. Periodical. of. Ocean. University. of. China. 2015; 45: 100–108.

[29] Zou GH, Cui JY, Liu ZL, Sun L. Simulating non-point pollution at watershed scales: a case study in Dagu watershed. Res. Sci. 2008; 30: 288–295.

[30] Li W, Cui LJ, Pang BL, Ma MY, Kang XM. Thinking of solving double counting in wetland ecosystem services valuation. Ecol. Environ. Sci. 2014; 23: 1716–1724.

[31] Li K, Cui LJ, Li W, Kang XM, Zhang YQ. Removing double counting in wetland ecosystem services valuation based on energy algebra. Chinese. J. Ecol. 2016; 35: 1108–1116.

[32] Chai J, Wang ZQ, Zhang HW. Integrated evaluation of coupling coordination for land use change and ecological security: a case study in wuhan city of hubei province, china. Int. J. Environ. Res. Public. Health. 2017; 14: 1435. doi: 10.3390/ijerph14111435 29165365 PMC5708074

[33] Cui SF. Combined simulation and prediction of surface water and groundwater in Daguhe river basin under changing environment. Shandong. Normal. University. Doctoral dissertation. 2015.

[34] Groot RS, Stuip M, Finlayson M, Davidson N. Valuing Wetlands: guidance for valuing the benefits derived from wetland ecosystem services. International. Water. Management. Institute. 2006.

[35] Liu D, Liu C, Fu Q. Construction and application of a refined index for measuring the regional matching characteristics between water and land resources. Ecol. Indic. 2018; 91:203–211.

[36] Saaty TL. A scaling method for priorities in hierarchical structures. J. Math. Psychol.1977; 15: 234–281

[37] Saaty TL. Decision making for leaders: the analytic hierarchy process for decisions in a complex world. European Journal of Operational Research 2013.42(1): 107–109

[38] Taheri K, Gutiérrez F, Mohseni H, Raeisi E, Taheri M. Sinkhole susceptibility mapping using the analytical hierarchy process (AHP) and magnitude–frequency relationships: A case study in Hamadan province, Iran. Geomorphology. 2015; 234: 64–79.

[39] Luu C, Meding J V, Kanjanabootra S. Assessing flood hazard using flood marks and analytic hierarchy process approach: a case study for the 2013 flood event in Quang Nam, Vietnam. Nat. Hazards. 2018; 90(1): 1–20

[40] Sun B D, Tang J C, Yu D H, Song Z W, Wang P G. Ecosystem health assessment: A PSR analysis combining AHP and FCE methods for the Jiaozhou Bay, China. Ocean. Coast. Manage.2019; 168: 41–50.

[41] Robledano-Aymerich F, Romero-Díaz A, Belmonte-Serrato F, Zapata-Pérez VM, Martínez-Hernández C, et al. Ecogeomorphological consequences of land abandonment in semiarid Mediterranean areas: integrated assessment of physical evolution and biodiversity. Agr. Ecosyst. Environ. 2014; 197: 222–242.

[42] Nie Y, Liu Q, Wang J, Zhang Y, Liu S. An inventory of historical glacial lake outburst floods in the Himalayas based on remote sensing observations and geomorphological analysis. Geomorphology. 2018; 308: 91–106.

[43] Li GM, Dong ZB, Sun H, Qian GQ, Luo WY, et al. Research on the value of ecosystem services of the North Plain of Henan province over the past 25 years. Res. Environ. Sci. 2010; 23: 1136–1141.

[44] Sutadian AD, Muttil N, Yilmaz AG, Perera BJC. Using the Analytic Hierarchy Process to identify parameter weights for developing a water quality index. Ecol. Indic. 2017; 75: 220–233.

[45] Xu W, Zhang X, Xu Q, Gong H, Li Q, Liu B. Study on the coupling coordination relationship between water-use efficiency and economic development. Sustainability. 2020; 12. doi: 10.20944/preprints202002.0085.v1

[46] Taylor CM, Harding RJ, Essery RLH. The influence of land use change on climate in the Sahel. J. Climate. 2002; 15: 3615–3629.

[47] Nakazato T, Warren DL, Moyle LC. Ecological and geographic modes of species divergence in wild tomatoes. Am. J. Bot. 2010; 97: 680–693. doi: 10.3732/ajb.0900216 21622430

[48] Fetahi T, Schagerl M, Mengistou S, Libralato S. Food web structure and trophic interactions of the tropical highland Lake Hayq, Ethiopia. Ecol. Model. 2017; 222: 804–813.

[49] Fang HW, Zheng Y, Zhang B, Han D, Tao YC. Decision-support system for flood control in Dagu River Basin in Qingdao. Advances in Science and Technology of Water Resources, 2008, 28(3):66–69.

[50] Yan DH, He Y, Wang H, Qin DY, Wang JH. Review of effect of the eco-hydrological process on water environment. Advances in Water Science, 2005, 16(5): 747–752.

[51] Zhang ZQ, Xiao Q, Wang CL. Analysis on comprehensive control mode of Dagu River Basin. Water Resources Development and Management, 2018, 35(12): 7–11.

[52] Liu JX, Yuan XL. Application of numerical modeling to prediction of water quality in Dagu River groundwater source area, Qingdao. Marine Geology Letters, 2006, 22(2): 9–14.

[53] Mamat Z, Halikümüt, Keyimu M, Keram A, Nurmamat K. Variation of the floodplain forest ecosystem service value in the lower reaches of Tarim river, china. Land. Degrad. Dev. 2017; 29: 47–57.

[54] Tzilivakis J, Warner DJ, Green A, Lewis KA. Spatial analysis of the benefits and burdens of ecological focus areas for water-related ecosystem services vulnerable to climate change in europe. Mitig. Adapt. Strat. Gl. Change. 2019; 24: 1–29.

[55] Collard SJ, Zammit C. Effects of land-use intensification on soil carbon and ecosystem services in brigalow (acacia harpophylla) landscapes of southeast Queensland, Australia. Agr. Ecosyst. Environ. 2006; 117: 185–194.

[56] Booth BBB, Jones CD, Collins M, Totterdell IJ, Cox PM, et al. High sensitivity of future global warming to land carbon cycle processes. Environ. Res. Lett. 2012; 7: 024002.

[57] Mcclellan M, Montgomery R, Nelson K, Becknell J. Comparing forest structure and biodiversity on private and public land: secondary tropical dry forests in Costa Rica. Biotropica. 2018; 50: 510–519.

[58] Vollmer D, Shaad K, Souter NJ, Farrell T, Dudgeon D, et al. Integrating the social, hydrological and ecological dimensions of freshwater health: the freshwater health index. Sci. Total. Environ. 2018; 627: 304–313. doi: 10.1016/j.scitotenv.2018.01.040 29426153

[59] Verones F, Huijbregts MA, Chaudhary A, De BL, Koellner T, et al. Harmonizing the assessment of biodiversity effects from land and water use within LCA. Environ. Sci. Technol. 2015; 49: 3584–3592. doi: 10.1021/es504995r 25719255

[60] Shen L, Huang Y, Huang Z, et al. Improved coupling analysis on the coordination between socio-economy and carbon emission. Ecol. Indic. 2018, 94: 357–366.

[61] Yang Y, Bao W, Liu Y. Coupling coordination analysis of rural production-living-ecological space in the Beijing-Tianjin-Hebei region. Ecol. Indic. 2020, 117(4): 106512.

[62] He J, Wang S, Liu Y, et al. Examining the relationship between urbanization and the eco-environment using a coupling analysis: Case study of Shanghai, China. Ecol. Indic. 2017, 77:185–193.

[63] Liu N, Liu C, Xia Y, et al. Examining the coordination between urbanization and eco-environment using coupling and spatial analyses: A case study in China. Ecol. Indic. 2018, 93:1163–1175.

