# Supplementary material for: Coupling coordination relationship between ecosystem services and water-land resources for the Daguhe River Basin, China
Source: PLoS One. 2021 Sep 10;16(9):e0257123. doi: 10.1371/journal.pone.0257123 (PMC8432845; doi:10.1371/journal.pone.0257123)
Supplement: S1 Table — (DOCX) [file pone.0257123.s001.docx]

**S1 Table. The economic values of different ecosystem services for eight regions in 2000**

**(Chinese Yuan: RMB)**

| **Districts** | Substance production | Carbon sequestration | Gas regulation | Climate regulation | Water purification | Leisure tourism |
| --- | --- | --- | --- | --- | --- | --- |
| Zhaoyuan | 1.21*10^6^ | 1.47*10^6^ | 1.91*10^6^ | 2.34*10^6^ | 0.29*10^6^ | 0.56*10^6^ |
| Pingdu | 1.82*10^6^ | 2.10*10^6^ | 2.65*10^6^ | 2.63*10^6^ | 0.32*10^6^ | 0.63*10^6^ |
| Jimo | 1.52*10^6^ | 1.47*10^6^ | 2.35*10^6^ | 2.04*10^6^ | 0.25*10^6^ | 0.40*10^6^ |
| Chengyang | 0.30*10^6^ | 0.53*10^6^ | 1.03*10^6^ | 0.58*10^6^ | 0.07*10^6^ | 0.17*10^6^ |
| Xihai’an | 0.81*10^6^ | 0.42*10^6^ | 0.88*10^6^ | 0.73*10^6^ | 0.09*10^6^ | 0.23*10^6^ |
| Gaomi | 0.91*10^6^ | 0.84*10^6^ | 1.18*10^6^ | 1.31*10^6^ | 0.16*10^6^ | 0.26*10^6^ |
| Laixi | 2.02*10^6^ | 1.89*10^6^ | 2.21*10^6^ | 2.63*10^6^ | 0.32*10^6^ | 0.63*10^6^ |
| Jiaozhou | 1.52*10^6^ | 1.79*10^6^ | 2.50*10^6^ | 2.34*10^6^ | 0.29*10^6^ | 0.43*10^6^ |
| Total | 1.01*10^7^ | 1.05*10^7^ | 1.47*10^7^ | 1.46*10^7^ | 0.18*10^7^ | 0.33*10^7^ |
