# Supplementary material for: Coupling coordination relationship between ecosystem services and water-land resources for the Daguhe River Basin, China
Source: PLoS One. 2021 Sep 10;16(9):e0257123. doi: 10.1371/journal.pone.0257123 (PMC8432845; doi:10.1371/journal.pone.0257123)
Supplement: S2 Table — (DOCX) [file pone.0257123.s002.docx]

**S2 Table. The economic values of different ecosystem services for eight regions in 2005**

**(Chinese Yuan: RMB)**

| **Districts** | Substance production | Carbon sequestration | Gas regulation | Climate regulation | Water purification | Leisure tourism |
| --- | --- | --- | --- | --- | --- | --- |
| Zhaoyuan | 1.54*10^6^ | 1.76*10^6^ | 2.34*10^6^ | 2.74*10^6^ | 0.37*10^6^ | 0.73*10^6^ |
| Pingdu | 2.30*10^6^ | 2.52*10^6^ | 3.24*10^6^ | 3.08*10^6^ | 0.41*10^6^ | 0.82*10^6^ |
| Jimo | 1.92*10^6^ | 1.76*10^6^ | 2.88*10^6^ | 2.39*10^6^ | 0.32*10^6^ | 0.52*10^6^ |
| Chengyang | 0.38*10^6^ | 0.63*10^6^ | 1.26*10^6^ | 0.68*10^6^ | 0.09*10^6^ | 0.22*10^6^ |
| Xihai’an | 1.02*10^6^ | 0.50*10^6^ | 1.08*10^6^ | 0.86*10^6^ | 0.12*10^6^ | 0.30*10^6^ |
| Gaomi | 1.15*10^6^ | 1.01*10^6^ | 1.44*10^6^ | 1.54*10^6^ | 0.21*10^6^ | 0.34*10^6^ |
| Laixi | 2.56*10^6^ | 2.27*10^6^ | 2.70*10^6^ | 3.08*10^6^ | 0.41*10^6^ | 0.82*10^6^ |
| Jiaozhou | 1.92*10^6^ | 2.14*10^6^ | 3.06*10^6^ | 2.74*10^6^ | 0.37*10^6^ | 0.56*10^6^ |
| Total | 1.28*10^7^ | 1.26*10^7^ | 1.80*10^7^ | 1.71*10^7^ | 0.23*10^7^ | 0.43*10^7^ |
