# Supplementary material for: Coupling coordination relationship between ecosystem services and water-land resources for the Daguhe River Basin, China
Source: PLoS One. 2021 Sep 10;16(9):e0257123. doi: 10.1371/journal.pone.0257123 (PMC8432845; doi:10.1371/journal.pone.0257123)
Supplement: S3 Table — (DOCX) [file pone.0257123.s003.docx]

**S3 Table. The economic values of different ecosystem services for eight regions in 2010**

**(Chinese Yuan: RMB)**

| **Districts** | Substance production | Carbon sequestration | Gas regulation | Climate regulation | Water purification | Leisure tourism |
| --- | --- | --- | --- | --- | --- | --- |
| Zhaoyuan | 1.68*10^6^ | 1.90*10^6^ | 2.56*10^6^ | 2.98*10^6^ | 0.42*10^6^ | 0.75*10^6^ |
| Pingdu | 2.52*10^6^ | 2.72*10^6^ | 3.55*10^6^ | 3.35*10^6^ | 0.47*10^6^ | 0.84*10^6^ |
| Jimo | 2.10*10^6^ | 1.90*10^6^ | 3.15*10^6^ | 2.60*10^6^ | 0.36*10^6^ | 0.53*10^6^ |
| Chengyang | 0.42*10^6^ | 0.68*10^6^ | 1.38*10^6^ | 0.74*10^6^ | 0.10*10^6^ | 0.22*10^6^ |
| Xihai’an | 1.12*10^6^ | 0.54*10^6^ | 1.18*10^6^ | 0.93*10^6^ | 0.13*10^6^ | 0.31*10^6^ |
| Gaomi | 1.26*10^6^ | 1.09*10^6^ | 1.58*10^6^ | 1.67*10^6^ | 0.23*10^6^ | 0.35*10^6^ |
| Laixi | 2.80*10^6^ | 2.45*10^6^ | 2.96*10^6^ | 3.35*10^6^ | 0.47*10^6^ | 0.84*10^6^ |
| Jiaozhou | 2.10*10^6^ | 2.31*10^6^ | 3.35*10^6^ | 2.98*10^6^ | 0.42*10^6^ | 0.57*10^6^ |
| Total | 1.40*10^7^ | 1.36*10^7^ | 1.97*10^7^ | 1.86*10^7^ | 0.26*10^7^ | 0.44*10^7^ |
